# Encephalitis, Mild Encephalitis, Neuroprogression, or Encephalopathy—Not Merely a Question of Terminology

**DOI:** 10.3389/fpsyt.2018.00782

**Published:** 2019-02-06

**Authors:** Karl Bechter

**Affiliations:** Department Psychiatry and Psychotherapy II, Bezirkskrankenhaus Günzburg, Ulm University, Ulm, Germany

**Keywords:** meningoencephalitis, encephalitis, neuroinflammation, inflammation, parainflammation

## Abstract

**Background:** Psychoneuroimmunology research has presented emerging evidence of the involvement of inflammatory and immune mechanisms in the pathogenesis of severe mental disorders. In this context, new terms with increasing clinical relevance have been proposed, challenging the existing terms, and requiring consensus definitions of the new ones.

**Method:** From a perspective of longstanding personal involvement in clinical settings and research in psychoneuroimmunology, the new and the existing terms are critically reconsidered.

**Results:** Meningoencephalitis and encephalitis are comparably well defined clinical terms in neuropsychiatry, although in the individual case approach diagnosis can be difficult, for example in some cases of encephalitis that are described with normal cerebrospinal fluid findings, or often in chronic encephalitis. Encephalopathy is also a widely accepted term, however, with a surprisingly broad meaning with regard to the assigned underlying pathophysiology, ranging from one-hit traumatic encephalopathy to inflammatory encephalopathy, the latter term addressing a type of brain dysfunction secondary to acute systemic inflammation without proven brain autochthonus inflammation (neuroinflammation). However, this latter assumption and term may be wrong as neuroinflammation is difficult to prove *in vivo*. With emerging insights into prevailing inflammatory and neuroinflammatory mechanisms that are involved in the pathogenesis of severe mental disorders, the interdependent aspects of sensitive assessment and potential clinical relevance of mild neuroinflammation are becoming more apparent and of increasing clinical interest. The new terms “mild encephalitis,” “parainflammation,” and “neuroprogression” show considerable overlap in addition to gaps and hardly defined borders. However, details are hard to discuss as available studies use many biomarkers, but most of these are done without an established categorical attribution to exclusive terms. Most important, the three new concepts (neruoprogression, parainflammation, and mild encephalitis) are not mutually exclusive, even at the individual case level, and therefore will require state-related individual assessment approaches beyond large confirmatory studies.

**Conclusion:** The newly proposed terms of mild encephalitis, parainflammation, and neuroprogression have an emerging clinical relevance, but respective borders, gaps and overlap in between them remain unclear, and these concepts may even be seen as complementary. Categorical delineation of the new and reconsideration of the existing terms with respect to individualized psychiatric treatment is required for better clinical use, eventually requiring a consensus approach. Here, a critique based on available data and a focus on clinical perspective was outlined, which may help to enhance fruitful discussion. The idea followed here is in line with pillar number six as proposed for the Research Diagnostic Domains, i.e., to provide and follow new concepts in psychiatric research.

## Introduction

Neuroinflammation, like inflammation in general, represents a dimensional or graded response embedded in time and space. Such a principle is even true in neurodevelopmental perspectives, recently exemplified for microglia (1). For clinical purposes, such a dynamic is split into separate categories, because categorization is helpful and needed to guide diagnostic management and especially appropriate treatment decisions. Inherent to such an approach is not only a partial loss of the dynamic perspective, but it poses also the new problem to define borders between neighboring categories as exactly as possible. However, categorical definition is based on actual theories and evidence and not least diagnostic methods available in the clinical approach. The paradigm of clinically relevant neuroinflammation represents infectious (meningo-)encephalitis, representing an acute, strong type of neuroinflammation, thus up to now also dominating the definition of terms beyond states of classical acute neuroinflammation. Suggestive or possible clinically relevant states of (mild) neuroinflammation not fulfilling the definition of meningoencephalitis, were not well classified or might sometimes even have been assigned as “non-inflammatory” diseases in clinical use and research (see below definition of neuroinflammation), with the terms mild neuroinflammation or parainflammation not in use. However, lower grades of neuroinflammation as compared to classical defined neuroinflammation might also be clinically relevant, especially when lasting over longer periods of time, but apparently are *a priori* more difficult to diagnose in individual cases and to categorize within a theoretical framework. The idea that mild encephalitis (ME) may be under-recognized though clinically relevant for severe mental disorders (SMI) (2), was not well accepted when first published in 2001 (3), but is gaining more support now (3–5). The discovery of NMDAR autoantibodies in 2007/8 by Josep Dalmau and his group and the since-emerging recognition and definition of Autoimmune Encephalitis (AE) for a widening spectrum of neurological disorders (4, 5) have especially moved the field, because AE can be well diagnosed as a result of presenting with severe neurologic symptoms. Most important, the early stages of AE are associated with various and varying, initially pure psychiatric syndromes, with neurological symptoms appearing only later in more severe stages, in addition to psychiatric syndromes (4, 5). Thus, mild neuroinflammation, or ME, can retrospectively be assumed to have been present during early stages of AE, as is evidenced from clinical course and by the later, more severe findings and symptoms categorized as AE. The emerging insight that some cases of psychosis may even represent previously undetected cases of AE (6, 7), led to an enhanced interest in improved and new diagnostic approaches to ME and a search for appropriate treatments in new-onset and therapy-resistant SMI. This was strongly reinforced by single case reports of successful immune modulatory treatments of cases of Autoimmune Psychosis (AP), with AP cases not fulfilling the diagnostic criteria of AE (8–13). Given the widely unexplained causality of SMI, one should recognize that ME in theory and practice appears sufficient and even prone to cause a spectrum of psychiatric symptoms even though it presents without neurological symptoms (at least without so-called neurological hard signs), and is thus within a spectrum of SMI (2). Therefore, the question of potentially prevailing but under-diagnosed ME in SMI is of great interest and relevance for improved treatments, including those with potentially rapid therapeutic success. Such a situation requires also a critical reconsideration of existing clinical terms in use, because more refined categorical classification is then required for research and clinical approaches. This is attempted here from a clinical and research.

## Critical Outline of Terms in Clinical use Around Neuroinflammation

### (Meningo-)Encephalitis

Encephalitis is used to term meningoencephalitis when involvement of meninges is apparent with respect to clinical symptoms and/or by findings (14). The most important diagnostic measure is CSF examination plus neuroimaging. Most diagnosed cases represent a type of acute severe encephalitis, with the disease sometimes being life-threatening.

Chronic encephalitis is overall rare. A clear definition of chronicity is difficult and a time frame of 4 weeks and beyond appears to be used by some in the clinical field, but a generally accepted sound definition of the term “chronic” was not to be found. Chronic encephalitis presents—with regard to the clinical picture—similarly to acute encephalitis, with the course just being protracted *a priori*, possibly with dominating or exclusive psychiatric symptoms in extended early stages of the disease. Respective psychiatric syndromes are typically unspecific, i.e., various and variant, though some symptom characteristics may be found, especially when including systemic signs and findings (14, 15).

Acute encephalitis is usually represented by severe acute brain inflammation possibly involving the brain and the meninges. Nevertheless, a theoretically sound definition is difficult (for example, exact delineation of encephalitis vs. with or without meningitis? Or is there always some co-occurrence?), whereas consensus on clinical case definition and diagnostic approach is well established (16). Of note is that in rare cases even CSF examination, the most sensitive diagnostic procedure followed by neuroimaging, may present with normal findings [Benninger and Steiner in Deisenhammer et al. (14), Venkatesan et al. (16)].

### Encephalopathy

The term encephalopathy appears to be broadly used, with a long tradition but with an apparent weakness of precision in its meaning. Encephalopathy was mainly used for lasting consequences of various insults to the brain, for example traumatic encephalopathy, vascular encephalopathy or epileptic encephalopathy. A recent case definition differentiates encephalitis from inflammatory encephalopathy, the latter addressing the case of brain dysfunction from severe systemic inflammation [compare with (16)]. In the context of the new developments in the field of psychoneuroimmunology the weakness of the existing term becomes clear; for example, the term “inflammatory encephalopathy” left open the question of possibly prevailing mild neuroinflammation beyond mere signaling effects in the brain from circulating cytokines and inflammatory markers, the latter mechanism assumed to underlie the “encephalopathy.” Also, with epileptic encephalopathy the problem becomes apparent; it is established that repeated epileptic seizures can lead to subtle damage of the brain, which can be prevented with good control of seizures, and such pathology would match, like traumatic encephalopathy, with the traded use of the term, typically addressing cases of lasting brain pathology from single or multiple hits. From a pathogenetic point of view, a completely different scenario represents a subgroup of epileptic disorders, which is consistent with rather new findings, which is caused by a subtype of AE and can be successfully treated with immune modulatory treatments (17, 18). The pathogenetic scenarios behind these two types of epileptic “encephalopathy” (when using the term encephalopathy in the broad sense as often found yet in actual literature) apparently differ strongly. The first one represents a state of defect, the second one a state of active neuroinflammation though clinically presenting nearly identically and appearing seemingly “non-inflammatory,” if not differentiated by the new diagnostic methods available for AE, and creating a new case group of AE presenting as epilepsy. To use the term encephalopathy for such differing pathogenetic scenarios is hardly acceptable, because such broad meaning of terminology is inappropriate to guide clinical decisions.

### Autoimmune Encephalitis (AE)

The only recently described subtype—AE—of encephalitis was mainly related to the discovery of CNS autoantibodies, found in blood and more sensitively in CSF, with cases presenting various neurological signs of encephalitis as established in clinical neurology (19–22). Most interestingly, the spectrum of neurological disorders associated with the prevalence of CNS autoantibodies is emerging since the discovery of NMDAR-autoantibodies, much beyond the previously-known cases of limbic encephalitis and tumor-associated paraneoplastic encephalitis, and emerging as well is the number of CNS autoantibodies discovered (23–30). However, many questions remain to be solved, especially with regard to the role of CNS autoantibodies in psychiatric disorders (27, 31–37).

### Mild Encephalitis (ME)

ME was proposed as a term in 2001 (3), placed categorically in between encephalopathy and encephalitis. Mild forms of encephalitis (or minor neuroinflammation) have been detected with careful histopathological investigation in certain disease phases of experimental Borna disease virus infection, a strongly neurotropic virus that causes classical-type meningoencephalitis in some species and in others presents just mild neuroinflammation paralleled by behavioral syndromes. Most important, the observed symptoms and course aspects over years (in animals) remembered variant symptomatology and courses of major psychiatric disorders in humans (3). Indeed, many findings including epidemiological and course aspects in affective and schizophrenic spectrum disorders would match with an ME scenario subgroup, just the limited sensitivity of available clinical methods may explain that diagnosis of ME cases failed in clinical reality (2, 3). Meanwhile, evidence is increasing that an ME subgroup indeed prevails in broadly defined affective and schizophrenic spectrum disorders [compare (8, 9, 38–43)]. In only very rare cases of subacute psychoses, performance of a brain biopsy was indicated for apparent ethical reasons; these rare though important studies demonstrated not only mild neuroinflammation in the cortex, but even more importantly, demonstrated good response to immune modulatory treatments (13, 38, 44). Given the rather high prevalence of minor CSF abnormalities in SMI groups (45–48), the size of an apparent prevailing ME subgroup in SMI patients remains to be carefully investigated.

### Autoimmune Psychosis (AP)

The term autoimmune psychosis was proposed recently (49) and taken up by other groups (8, 9). The term AP could well make sense, despite a yet-limited precision of its definition in a pathogenetic perspective. Primarily addressing the presence of CNS autoantibodies [compare with (49)] or, in an extended definition of AP, some apparent clinical plausibility of a prevailing autoimmune process [compare (9)], does not convincingly demonstrate the assumed neuroinflammatory mechanisms behind psychosis, except in cases with respective findings in brain biopsy or CSF or neuroimaging. Nevertheless, respective criteria of a consensus diagnosis of AP are just being developed. It seems that the problem of diagnosis/definition of AP (dependent on the methods used and available, see above) is rather similar to the problem with the term and definition/diagnosis of “inflammatory encephalopathy,” leaving major open questions about the categorical borders between parainflammation (see below) vs. mild neuroinflammation vs. classical neuroinflammation.

### Parainflammation

The term parainflammation represents a new definition for low-grade inflammation (50), recently adapted for mental health disorders and exemplified for “stress-induced parainflammation” by Wohleb (51): Wohleb proposed a situation similar to general parainflammation as proposed by Medzhitov to represent the underlying pathophysiological mechanism in some mental health disorders, a mild form of inflammation assigned as parainflammation of the brain when being inducible and induced by systemic inflammatory activation, especially stress. Parainflammation would be characterized by evolving neuroimmune processes and microglial changes, with the altered microglia-neuron interactions eventually leading to neuroplasticity deficits and neuronal dystrophy. The (para-)inflammatory changes from homeostasis were considered modest in quality and degree as compared to changes which defined neuroinflammation. CNS-related parainflammation was discussed to play a role in mental health disorders like anxiety and depressive-like behavior in aging and in neurodegenerative disorders. Criteria to define stress-induced parainflammation of the CNS appeared however difficult to establish and reliably reproduce, if not in the stress paradigm. The respective claimed borders to neuroinflammation appear rather unclear or arbitrary and must be worked out. The relation of CNS parainflammation to neuroprogression remained open, but conceptual overlap apparently exists. Nevertheless, the concept of CNS parainflammation appears to represent another valuable attempt to tackle the problem of a refined grading of immune-inflammatory responses in the CNS in clinically useful categories.

### Neuroinflammation (NI)

The definition of NI differs in details between different fields (virology, histopathology, clinical field, others) due to the differing assessment methods used in respective fields. Therefore, a simple translation of the definition of NI from one field to the other is complicated and, for evident reasons, the case of mild chronic NI is more problematic as compared to severe acute NI. A general problem of the clinical definition of NI relates to the limited approach to the brain in clinical patients; even the diagnosis of severe acute NI requires the use of a combination of various methods, each with an overall limited sensitivity to detect NI at the individual patient level. The most relevant diagnostic methods herein are represented by CSF examination followed by neuroimaging. The most sensitive method in principle represented brain biopsy, but its application is strongly limited due to apparent ethical reasons and, even when indicated, from the difficulty of choosing the appropriate brain site for taking the biopsy, the latter aspect reducing its *a priori* high sensitivity. A critical review of the theoretical and practical gaps in defining NI in the various fields dealing with NI is hardly found in the literature.

An indirect example to critically review the clinical definition of NI can be found with actual recommendations for the use of CSF in biomarker studies (52): definitions and names of control groups (overall 6 groups) are as follows: Healthy Controls, Spinal Anesthesia Subjects, Central Inflammatory Neurologic Disease Controls, Peripheral Inflammatory Neurologic Disease Controls, Noninflammatory Neurologic Disease Controls, Symptomatic Controls. The defining criteria of each group are based on multilevel clinical findings, including exclusion criteria by CSF findings. The use of CSF findings to define controls apparently represents in some way a circular argument for definition, but to find better options for definition is difficult, at least not available, because completely healthy controls are not only hard to find as controls in studies requiring invasive methods, but is also hardly justified to use controls in all studies. The compromises needed to deal with the problem of controls in studies may, however, posit a major problem for studies about mild neuroinflammation, although less so with classical severe acute neuroinflamamtion. Herein, the most apparent weak aspect in defining categories/subgroups of controls was the CSF criteria for non-inflammatory neurologic disorders (cell count must be normal, QAlb can be normal or elevated). The idea of mild neuroinflammation appears, at least to me, still poorly understood, given the high frequency of minor CSF abnormalities in SMI groups (see also above). One should consider that normal values for CSF parameters were established in clinical neurology, which from apparent circumstantial factors may cause a preference for observing severe neurological disorders only and, in the case of NI, a preference for observing only cases with severe neuroinflammation. This may lead to missing milder forms of NI or even missing cases of severe meningoencephalitis, which can be observed with normal CSF numbers (see above). Therefore, a justified conclusion is that available methods, including even the major pillars of NI diagnostics in the clinic, CSF examination and neuroimaging, are likely, and by many experts admittedly, insensitive for an individualized diagnosis of mild NI.

### Neuroprogression

The term neuroprogression, proposed by Berk et al. (53), refers to a combination of treatment non-responsiveness, relapsing and declining course of illness, and progressive neuropathological changes commonly seen in several psychiatric disorders (54). The concept of neuroprogression is emerging and well supported (55–58). To establish defining criteria of neuroprogression in an individualized approach appears, however, presently unsolved, because neuroprogression involves many pathogenic mechanisms including microglial activation, inflammation and systemic toxicity (59). Nevertheless, for clinical use, eventually a categorical definition will be required to guide appropriate therapeutic approaches at the individual case level (60). The theory appears yet fully informative for select testing of respective treatment approaches in study design, which does not appear to be the case for individual treatment approaches like with the ME hypothesis (see above). The problems of overlap and unclear categorical differences between neuroprogression, parainflammation, and mild neuroinflammation, also discussed in context with AP and inflammatory encephalopathy, are apparent, but all these concepts have innovative power. A crucial question herein is whether, how and when, which one of the three newly introduced concepts (meaning in principle more or less strong active states of neuro-immuno-inflammation) may be relevant at an individual case level and in the course of the disease.

### Relation to Research Domain Criteria (RDoC) Research

The development of RDoCs under the guidance of the National Institutes of Health to improve the basics of available diagnostic systems in psychiatry is driven by limited progress in psychiatric therapies from psychiatric research; to overcome this frustrating situation new approaches are claimed to be grounded on seven new pillars (61). Along this new line of orientation of psychiatric research, the sixth pillar is approached here; that is, a new conceptualization, respectively constructs with immediate relevance for new individualized treatments. An emerging collection of single cases demonstrates the utility of the ME concept in severe SMI at the individual case approach of selected cases. The new concepts of parainflammation and of neuroprogression may be of more general relevance. Parainflammation may act as a contributive or triggering factor in severe mental disorders and as a causative factor for minor psychiatric disorders as presented with the still theoretical concept, which is able to explain a number of established findings as at least one possible interpretation; neuroprogression appears to be relevant to understand the long-term aspects and consequences from lasting neuroinflammatory dysregulation in rather large subgroups of SMI, a conclusion that is backed by a number of emerging studies to support the concept with good theoretical plausibility. Nevertheless, this evaluation of these two concepts does not disregard the possibility that the concept of ME is appropriate even in a larger subgroup of SMI. The three new concepts may be relevant in a complementary perspective in general and over time during the course of SMI illness.

Arguments for the relevance of the ME concept are as follows: observed single cases of therapy-resistant schizophrenia-like syndromes (but diagnosed before as schizophrenia) or of severe major depression of rapid and often full remission (conclusion: cases were seemingly chronic, but in retrospect better considered subacute or mild NI) under various immune modulatory treatments including CSF filtration (62, 63); brain biopsy-proven ME in major depression (13); cortisone or antiepileptics (64–66); complex immune treatment with refined diagnosis of systemic autoimmune disorder underlying the psychiatric syndrome (seeming to be of a chronic-inactive character but in retrospect rather representing a subacute or waxing-waning immune-inflammatory pathology) (12); or a differential diagnosis of a difficult-to-detect agent related autoimmunity—that is, persistent infection-related autoimmunity, treatable by antibiotics and/or immune modulatory measures (11, 67–69).

## Conclusion

The traded clinical terms dealing with neuroinflammatory and other brain damaging disease states like neuroinflammation, (meningo-)encephalitis and encephalopathy should be reconsidered in light of recent developments in neuropsychoimmunology and completed by new terms. With new insights into the frequent prevalence of milder forms of neuroinflammation in severe mental disorders and in a variety of neurodegenerative disorders including dementia, parkinsonism and others not discussed in this review, a detailed categorical terminology of neuroinflammatory and neuroimmune mechanisms with respective clinical relevance would be helpful. Establishing refined new terms is however difficult due to many problems inherent to the diagnostic approach to the individual patient and the slowly emerging insights about the possible pathogenic relevance of new findings indicating some neuroinflammatory or neuroimmune aberrances. The key question about any potential relevance of identified factors in the pathogenesis of the respective disorder (causal or contributive factor, relative weight of single factor), is difficult to evaluate *a priori*. However, this is complicated by uncertainties about the sensitivity and limits of diagnostic methods available, the latter aspect directly influencing the validity of clinical definitions, which becomes a circular problem. For example, although prevalence of some neuroinflammatory activation was well established in the pathogenesis of dementia, the respective inflammatory CSF markers appear to be less important compared to metabolic biomarkers (70), thus raising the question of the weight of neuroinflammatory changes in respective disease pathogenesis. The newly proposed terms Mild Encephalitis, Neuroprogression, Stress-induced Parainflammation and Autoimmune Psychosis appear of special relevance for research on severe mental disorders, although they may yet remain difficult or impossible to assess with available clinical methods in an individual patient approach. However, also in theoretical framework and with respect to categorical borders of one term to another, overlap and gaps of knowledge have to be recognized and solved, but the new terms may set the stage for further research and development. With regard to existing terminology, the new terms can bridge the most apparent gaps in the clinical approach to the dimensional and quality aspects of (neuro-)inflammation by addressing potentially relevant though overall mild forms of neuroinflammation in the pathogenesis of severe mental disorders and in minor stress-induced mental disorders, thus they might importantly contribute to progress in psychiatric research and clinical settings. Teaching examples like the psychosomatics of gastric/duodenal ulcers vs. the bacterial triggered dysfunction/inflammation might be kept in mind. The term encephalopathy appears to be rather weakly defined, often used to assign various seemingly brain-diseased states in general medicine, neurology, and psychiatry. A most appropriate use of the term encephalopathy might represent disease states “after” some insult to the brain, like post-traumatic or post- encephalitic states, or in the case of preliminary diagnosis or of a scientifically unclear weight of identified single pathogenic factors within a complex brain disease pathogenesis. The term inflammatory encephalopathy appears rather questionable and should represent a focus of further research regarding the underlying brain pathology and detailed pathogenesis behind it [compare (16)]. A rather important step in such a direction was to perform CSF examination more frequently than presently done in the psychiatric field (59) and by advanced and newly developed diagnostic methods (71, 72). Such an approach follows the RDoC initiative assumed to bring psychiatric individualized treatments forward into a new, more successful level.

## Author Contributions

The author confirms being the sole contributor of this work and has approved it for publication.

### Conflict of Interest Statement

The author declares that the research was conducted in the absence of any commercial or financial relationships that could be construed as a potential conflict of interest.
